# Zoonotic helminths of dogs and risk factors associated with polyparasitism in Grenada, West Indies

**DOI:** 10.1017/S0031182023000495

**Published:** 2023-07

**Authors:** Maxine L.A. Macpherson, Patsy A. Zendejas-Heredia, Wayne Sylvester, Robin B. Gasser, Rebecca J. Traub, Vito Colella, Calum N.L. Macpherson

**Affiliations:** 1 Windward Islands Research and Education Foundation, St. George's, Grenada, West Indies; 2 School of Veterinary Medicine, St. George's University, St. George's, Grenada, West Indies; 3 Faculty of Science, Melbourne Veterinary School, University of Melbourne, Parkville, VIC, Australia

**Keywords:** *Ancylostoma*, dogs, hookworm, soil-transmitted-helminths (STHs), *Strongyloides*, *Toxocara*, *Trichuris*, polyparasitism

## Abstract

Canine soil-transmitted helminths (STHs) cause important zoonoses in the tropics, with varying degrees of intensity of infection in humans and dogs. This study aimed to investigate the prevalence and associated risk factors for STHs in community dogs residing in Grenada, West Indies. In May 2021, 232 canine fecal samples were examined for zoonotic helminths by microscopy (following flotation), and genomic DNA from a subset of 211 of these samples were subjected to multiplex qPCR for the detection and specific identification of hookworms, *Toxocara* spp. and *Strongyloides*. Microscopic examination revealed that 46.5% (108/232, 95% CI 40–52.9), 9% (21/232, 95% CI 5.35–12.7) and 5.2% (12/232, 95% CI 2.3–8) of the samples contained eggs of *Ancylostoma* spp., *Toxocara* spp. and *Trichuris vulpis*, respectively. Multiplex qPCR revealed that, 42.2% (89/211, 95% CI 35.5–48.8) were positive for at least 1 zoonotic parasite. Of these, 40.8% (86/211, 95% CI 34.1–47.3) of samples tested positive for *Ancylostoma* spp., 36% (76/211, 95% CI 29.5–42.9) were positive for *A. caninum,* 13.3% (28/211, 95% CI 9–18.6) for *A. ceylanicum*, 5.7% for *T. canis* (12/211, 95% CI 2.97–8.81) and 1% (2/211, 95% CI 0–2.26) for *Strongyloides* spp. (identified as *S. stercoralis* and *S. papillosus* by conventional PCR-based Sanger sequencing). Using a multiple logistic regression model, a low body score and free-roaming behaviour were significant predictors of test-positivity for these parasitic nematodes in dogs (*P* < 0.05). Further studies of zoonotic STHs in humans should help elucidate the public health relevance of these parasites in Grenada.

## Graphical abstract

**Figure:**
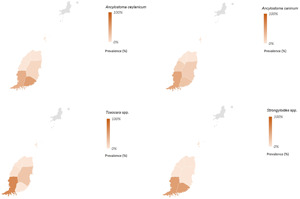

## Introduction

Dogs are the oldest companion animals of humans, and we share over 60 of the >400 infectious diseases that dogs can carry (Macpherson *et al*., 2022). These include zoonotic gastrointestinal parasites which are often of significant public health importance. In tropical regions of the world, such as in Grenada, West Indies, some of these have been reported, including *Ancylostoma caninum* and *A. ceylanicum* (Zendejas-Heredia *et al*., 2022). Dogs are the main reservoir hosts for these zoonotic parasites, due to their free roaming behaviour, uncontrolled reproduction and limited access to veterinary care (Lyons *et al*., 2022).

*Ancylostoma ceylanicum* and *Strongyloides stercoralis* are the only 2 known canine zoonotic soil transmitted helminths (STHs) that result in patent infections in humans (Schar *et al*., 2013, Bradbury *et al*., 2017). Currently, *A. ceylanicum* is recognized as the second most common species of hookworm infecting humans in the Asia Pacific region, comprising between 6–23% of all intestinal hookworm infections in people living in endemic areas (Traub *et al*., 2021). *Ancylostoma ceylanicum* and *A. caninum* can cause cutaneous larva migrans (CLM) and intestinal symptoms in humans, and haemorrhagic diarrhoea and chronic iron deficiency anaemia in dogs (Traub *et al*., 2021). The global prevalence of strongyloidiasis was estimated at 8.1% in 2017, with 76% of cases recorded in Southeast Asia, Africa and the Western Pacific (Buonfrate *et al*., 2020). *Strongyloides* spp. have the potential for transmission to humans and other animals through the parasite's ability to reproduce in the environment – a unique strategy amongst parasitic helminths of mammals (Jaleta *et al*., 2017). *Strongyloides* spp. infections in both dogs and humans are often asymptomatic but can become fatal together with chronic comorbidities (Streit, 2021). *Toxocara canis* is estimated to infect >100 million dogs worldwide (Rostami *et al*., 2020), with majority of puppies becoming infected through vertical transmission (Schwartz *et al*., 2022). Nonetheless older dogs can harbour patent infections as a consequence of a compromised immune status (Fahrion *et al*., 2008). Humans are often asymptomatic but can develop visceral larva migrans, ocular larva migrans, neurotoxocariasis and covert toxocariasis (Macpherson, 2013). A recent seroprevalence study showed that 37% (93/253; 95% CI 36–38) of 253 people in the local population were seropositive for *T. canis* using an IgG-based ELISA test (Ziegler, 2022, unpublished results), suggesting that humans are exposed to this parasite in Grenada. While initial treatment of puppies with an anthelmintic has been recommended at 2 weeks postpartum for decades, puppies in Grenada have been shown to already be shedding eggs at this time (Schwartz, *et al*., 2022).

There is a paucity of information on STHs in the Caribbean, including Grenada, which represents a major gap in the knowledge of the transmission and public health relevance of these parasites in this region. Thus, assessing factors involved in the transmission of these helminths should aid in developing targeted control programmes using a *One Health* approach (Walker *et al*., 2023).

Here, combined microscopic and molecular tools were used to estimate the prevalence and to molecularly identify nematodes affecting dogs in Grenada, and a multiple logistic regression model was used to assess risk factors associated with polyparasitism in these dogs.

## Materials and methods

### Ethics approval

This project was approved by the Institutional Animal Care and Use Committee (IACUC) of the St. George's University, Grenada, West Indies (approval code: IACUC-21001-R).

### Study area and population

Grenada is part of the Windward Islands in the Caribbean and has a land area of 348.5 km^2^. The human and dog populations are ~110 000 and 35 000, respectively (Catan and Macpherson, 2007).

In May 2021, fecal samples were obtained from community-owned and pet dogs throughout all 6 parishes, mainly from the southern half of the country. Many of these were Pothounds, which are nondescript, medium–sized dogs found throughout the Caribbean and other tropical areas (Catan and Macpherson, 2007). Pothounds are often loosely owned within a community and roam freely. They usually do not have a history of previous veterinary examination and are not regularly given drugs to control endo- or ecto-parasites (Schwartz *et al*., 2022).

### Sample collection

Most dogs in Grenada have a primary owner, even if they roam and are largely ‘community-owned’. This person was the point of contact for each dog. Following informed consent from dog owners, a physical examination was performed before fecal collection to record the body condition score (BCS) and general health status (e.g. anaemia or pregnancy status) of each dog sampled. Age was assessed from owner knowledge and also looking at age related changes such as the state of a dog's teeth. The American Animal Hospital Association (2010) 9 point body condition scoring system was used to guide BCS assignment for dogs enrolled in this study. Pregnancy was assessed upon history taking and physical examination and confirmed by a veterinarian using a portable ultrasound. Anaemia was assessed through mucous membrane colour. All physical examinations were performed by a third-year veterinary student under the supervision of an experienced clinical veterinarian. A minimum of 1–3 grams of feces was collected, except for young puppies where less than 1 g was taken. Fecal samples were obtained rectally or an aliquot was taken from the top of a fresh fecal pile immediately following observation of defecation. Each sample was accompanied by a written record of the date and time of sample collection, a name or description of identifying features of the dog, their sex and age, the village and parish of origin, whether it was allowed to roam or kept indoors, any history of preventative drug use, ectoparasites detected, and ‘owner’ details, including name and phone number. This information was stored in an Excel spreadsheet. Fecal samples were placed into individual sealed and labelled plastic tubes and stored in an insulated container containing ice packs. Samples were carried for 1–10 h during field collection before arriving in the laboratory, after which they were kept at 4°C. Aliquots (~1 g) of individual fecal samples were fixed in 70% ethanol and sent to The University of Melbourne (Australia) for the identification of species of STHs by multiplex qPCR (methodology described below).

### Microscopic examination of fecal samples

A conventional passive fecal flotation method (Dryden *et al*., 2005) was used for the detection of eggs. In brief, 0.5–1 gram of each fecal sample was homogenized in zinc sulphate solution (specific gravity: 1.2) in an Ovassay Plus fecal flotation device (Zoetis, USA) and a coverslip placed on the meniscus. After 15 min, the coverslip was lifted, inverted and placed on to a glass slide and examined at 10-times and 40-times magnification using light microscopy (Olympus CH30, USA). The presence and number of nematode eggs visualized under each entire cover slip was recorded.

### Extraction of DNA from fecal samples

DNA was extracted from aliquots of individual fecal samples (*n* = 220; ~200 mg each; following the removal of ethanol and sample rehydration) using a QIAmp® PowerFecal Pro DNA Kit (Qiagen, Germany) according to the manufacturer's instructions with slight modifications: (i) the cell lysis step was performed using 800 *μ*L of solution CD1 employing a FastPrep-24™ 5 G homogeniser (MP Biomedicals); and (ii) DNA was eluted twice using 50 *μ*L of solution C6, the 2 eluates combined and the samples then stored at −20°C until further processing.

### Multiplex qPCR

Individual DNA samples were tested (in duplicate) using multiplex Taq-Man probe-based qPCR (M-qPCR) assays to detect a partial region of the first internal transcribed spacer of nuclear rDNA region (ITS-1) of *A. caninum, A. ceylanicum, A. braziliense* and *U. stenocephala* (Massetti *et al*., 2020, Zendejas-Heredia *et al*., 2022), a partial region of the 18S rRNA gene of *Strongyloides* spp. (Verweij *et al*., 2009), and a partial region of the ITS-2 for *T. canis* (Durant *et al*., 2012). Equine herpes virus (EHV4) was used as an internal control within each PCR, and a mammalian target of a 92 bp–region of the mitochondrial 16S rRNA gene of mammals was employed as a DNA–extraction control (Table 1). M-qPCR reactions were performed as described previously using a modification of the Quantinova Probe PCR (Qiagen, Germany) conducted in a final volume of 10 *μ*L. The cycling conditions were 95°C for 2 min, followed by 40 cycles at 95°C for 15 sec and at 60°C for 1 min using the QIAquant 96x real-time PCR instrument (Qiagen, Germany). Synthetic DNA fragments (gBlocks Gene Fragments, IDT Technologies, Skokie, Illinois, USA) were used as positive controls, and a no-template (negative) control was included in each PCR run.

**Table 1.** Oligonucleotides, probe sequences and cycling conditions used for the multiplex qPCR reactions for differentiation of hookworm species and detection of *Strongyloides* spp.

Target Species	Oligos and Probes	Sequences 5*’*-*’*3	qPCR conditions	Gene target	Size (bp)	Ref
*A. ceylanicum* *A. caninum*	A. canceyF A. cancey R	GGG AAG GTT GGG AGT ATC G CGA ACT TCG CAC AGC AAT C	92°C/2 min x1 95°C/10 sec 60°C/60 sec x40	ITS-1	100	(Massetti *et al*., 2020)
	AceyDOG Probe Acan Probe	/Cy5/CCGTTC+CTGGGTGGC/3lAbRQSp /5HEX/AG+T+CGT+T+A+C+TGG/3IABkFQ				
*A. braziliense* *U. stenocepahala*	UncbrazF Unbraz R	GAGCTTTAGACTTGATGAGCATTG GCAGATCATTAAGGTTTCCTGAC			119 118	
	Abra Probe Unc Probe	56FAM/TGAGCGCTA/ZEN/GGCTAACGCCT/3IABkFQ/ 5HEX/CATTAGGCG/ZEN/GCAACGTCTGGTG/3IABkFQ				
*Strongyloides spp.*	STR F STR R	CCAAGTAAACGTAAGTCATTAGC CGCCTCTGGATATTGCTCAGTTCC		18S rRNA	101	(Verweij *et al*., 2009)
	STR Probe	5Cy5/ACACACCGG/ZEN/CCGTCGCTGC/IBFQ				
*Toxocara canis*	T canis P	5’-FAM-CCATTAC CACACCAGCATAGCTCACCGA -3’-BHQ1		ITS-2	141	(Durant *et al.* 2012)
	Tcan-F Tox R	5’-GCGCCAATTTATGGAATGTGAT-3 5’-GAGCAAACGACAGCSATTTCTT-3’’				
*Canine DNA control*	MAMF MAMR	CGACCTCGATGTTGGATCAG GAACTCAGATCACGTAGGACTTT		16S MtrRNA	92	(Massetti *et al*., 2020, Hii *et al*., 2018)
	MAM Probe	FAM/CCTAATGGT/ZEN/GCAGCAGCTATTAA/ LABKFQ				
*Equine Herpes Virus*	EHV-FWD EHV-REV	GATGACACTAGCGACTTCGA TTTCGCGTGCCTCCTCCAG		Gb	81	(Bialasiewicz *et al*., 2009)
	EHV Probe	ROX/TTTCGCGTGCCTCCTCCAG/3IAbRQSp				

### Conventional PCR and DNA sequencing

Samples shown to be test-positive for *Strongyloides* and *T. canis* by qPCR were subjected to conventional PCR (cPCR) and DNA sequencing for specific identification. A ~255 bp region of the nuclear 18S rRNA hypervariable region IV was selected as target to identify *Strongyloides* using the primers New_HVR_IV F (5’-CGGGCCGGACACTATAAGG-3’) and New_HVR_IV R (5’-ATCTCTAAACAGGAACATAATGATCACTAC-3) (Barratt *et al*., 2019). A ~300 bp partial region of the nuclear ITS-2 rRNA gene was selected to identify *T. canis* (Li *et al*., 2007). using primers YYI (5′-CGGTGAGCTATGCTGGTGTG-3′) and NC2 (5′-TTAGTTTCTTTTCCTCCGCT-3) (Li *et al*., 2007) The PCR conditions were 95°C for 5 min, followed by 40 cycles at 94°C for 30 sec, 63°C (*Strongyloides* spp.) or 56°C (*T. canis*) for 30 sec, 72°C 30 sec, followed by a final extension at 72°C for 5 min. The cPCR was conducted using HotStartTaq Plus DNA Polymerase (Qiagen, DEU) employing a SimpliAMP Thermal Cycler (Thermo Fisher Scientific, US). PCR products were examined on a 1.5% (w/v) agarose gel containing GelRed nucleic acid stain (Gene Target Solutions, AUS) using Tris/Borate/EDTA (TBE) buffer. Amplicons presenting as single bands of the expected size were individually treated with ExoSAP-IT PCR Product Cleanup Reagent (Thermo Fisher Scientific, USA) and subjected to Sanger sequencing. Sequences were examined using Geneious Prime® 2021.2.2 and matched to data in the GenBank database using the BLASTn algorithm.

### Statistical analyses

Prevalence data was analysed and displayed in the RStudio Team 2020 Integrated Development Environment for R (RStudio, PBC, Boston) and Microsoft Excel 2021; 95% confidence intervals (CIs) were calculated using the Wilson score interval *via* the open-source software Epitools (https://epitools.ausvet.com.au). Multiple logistic regression analyses were performed to infer associations with infection with gastrointestinal parasites, *Toxocara*, and hookworms with variables age, sex, BCS, lactation, health abnormalities, roaming behaviour and whether dogs had previously received an endoparasiticide, in Prism 9 (San Diego, CA, USA). A strategy of iterative backward-elimination was used to arrive at the final models. In each iteration, the explanatory variable that had the largest *P* value of those exceeding a threshold *α* *=* 0.2 was removed, and a new model was fitted. Associations between explanatory variables and the response variable were considered statistically significant if their *P* value was <0.05.

## Results

**Figure 1.**
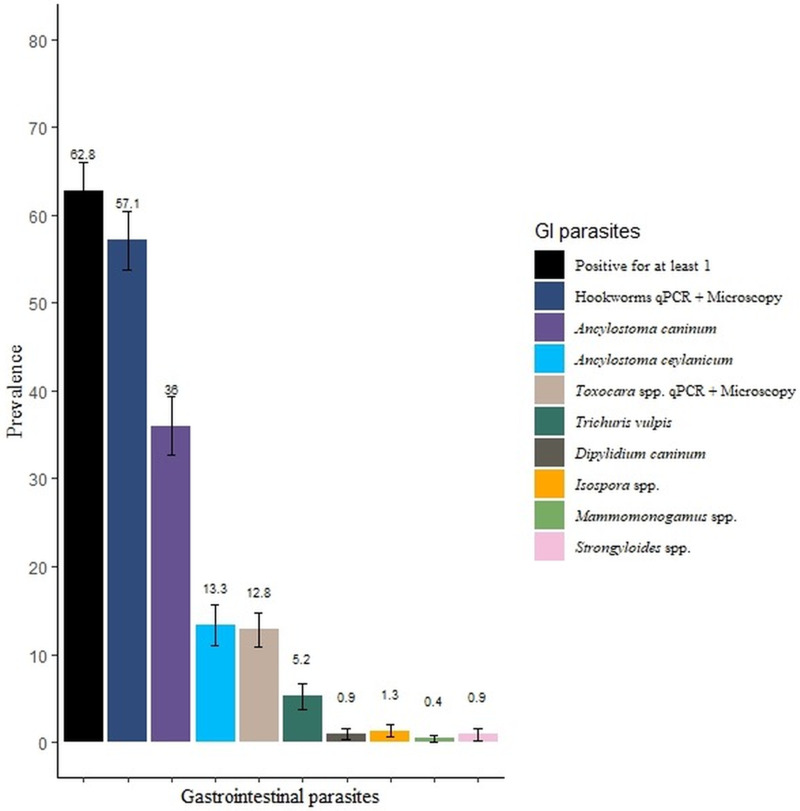
Prevalence of gastrointestinal parasites detected by microscopy and multiplex qPCR assays. Error bars indicate standard error mean.

In total, 232 fecal samples were collected from dogs ranging in age from 2 weeks to 15 years; 93 and 139 dogs were <1 and >1 year/s of age. Microscopic examination revealed eggs of *Ancylostoma* spp. in 46.5% (108/232, 95% CI 40–52.9), *Toxocara* spp. in 9% (21/232, 95% CI 5.35–12.7) and *T. vulpis* in 5.2% (12/232, 95% CI 2.3–8) of fecal samples (Table 2, Fig. 1).

**Table 2.** Number of animals <1 or >1 year of age positive for gastrointestinal parasites at microscopy (*) or qPCR (^#^) in 232 dogs from Grenada

Test positivity	<1 year	>1 year	Total
*Ancylostoma* spp.*	42	66	108
Toxocara canis*	18	3	21
Trichuris vulpis*	0	12	12
*Ancylostoma caninum*^#^	34	40	74
*Ancylostoma ceylanicum^#^*	6	22	28
*Strongyloides* spp.^#^	0	2	2

Polyparasitism with STHs was recorded in 14% (19/137, 95% CI 9–21) of positive samples, of which 12 were positive for *Ancylostoma* spp. and *Toxocara* spp., 6 for *Ancylostoma* spp. and *T. vulpis*, and 1 for all 3 parasites. An additional 2 samples contained *Ancylostoma* spp. and *Isospora* spp. and 1 contained *Ancylostoma* spp. and *Dipylidium caninum* proglottids. Occasionally other parasites were found, including 3 separate samples positive only for *D. caninum* egg packets, *Demodex* spp. and *Mammomonogamus* spp..

In total, 211 of the 220 samples contained a sufficient amount of starting material for molecular analysis based on an assessment of the Ct values for individual samples using DNA-extraction control and were subjected to M-qPCR. Out of the 211 samples, 42.2% (89/211, 95% CI 35.5–48.8) were positive for at least 1 zoonotic parasite. Of these, 40.8% (86/211, 95% CI 34.1–47.3) were test-positive for *Ancylostoma* spp., of which 36% (76/211, 95% CI 29.5–42.9) were positive for *A. caninum* and 13.3% (28/211, 95% CI 9–18.6) for *A. ceylanicum*, 5.7% (12/211, 95% CI 2.97–8.81) were positive for *T. canis*, and 1% (2/211 95% CI 0–2.26) for *Strongyloides* spp. ((Zendejas-Heredia *et al*., 2022; Table 2). *Uncinaria stenocephala* and *A. braziliense* positive samples were not detected by M-qPCR (Zendejas-Heredia *et al*., 2022). Two samples were positive by M-qPCR for *Strongyloides* spp.; Sanger DNA sequencing showed for one of them a 99% nucleotide identity with publicly available sequences for *S. stercoralis* (GenBank Accession nos. KU724129, MN076383 and MK468672; haplotype B corresponding to the *S. stercoralis* dog cluster; cf. Barratt *et al*., 2019; the other sequences showed a 100% nucleotide identity to sequences of *Strongyloides papillosus* (AB923886 and LM525870). In addition, sequencing of the ITS-2 of *Toxocara*-positive microscopy samples showed 100% nucleotide identity with *T. canis* sequences available in GenBank (accession nos. OM87669-73, OK635791-92, MK728991-2 and MH044068-73). Sequences obtained for *Strongyloides* spp. and *T. canis* are available in GenBank under accession nos. OQ862314-15 and OQ909098 and OQ909099, respectively.

A multiple logistic regression model identified the variables BCS [OR 1.19 (95% CI: 1.03–3.58), *P* = 0.04] and the status of free-roaming dogs [OR 2.39 (95% CI: 1.38–4.16), *P* = 0.002] as significant predictors of test-positivity to at least 1 gastrointestinal parasite (Table 2). Evidence of an association between free-roaming dogs [OR 1.9 (95% CI: 1.10–3.31), *P* = 0.004] and hookworm infection and BCS [OR 0.08 (95% CI: 0.004–0.42), *P* = 0.017] and *Toxocara* spp. infection was identified (Table 3).

**Table 3.** Parameter estimates and odds ratios (95% profile likelihood) for positivity to at least 1 gastrointestinal (GI) parasites and hookworms in 232 dogs from Grenada

Parameter estimates	Estimate	Standard error	Odds ratios	*P* value
At least 1 GI parasites				
Intercept	0.1152	0.2164		
Free roaming	0.8694	0.2811	2.39 (1.38–4.16)	0.002
Body condition score (BCS)	0.6431	0.3144	1.9 (1.03–3.58)	0.04
Hookworms				
Intercept	−0.8835	0.2135		
Free roaming	0.6423	0.2794	1.90 (1.10–3.31)	0.004
*Toxocara* spp.				
Intercept	−1.822	0.229		
Body condition score (BCS)	−2.455	1.033	0.08 (0.004–0.42)	0.017

## Discussion

Several zoonotic nematodes were reported as being endemic in the canine population of Grenada and both canine roaming behaviour and health status were identified as predictors for parasitism by multiple gastrointestinal nematodes. Using molecular tools, *S. stercoralis* and *S. papillosus* DNA were detected in individual dogs. The finding of *S. papillosus* is likely pseudoparasitism due to coprophagy of ruminant feces by dogs. The genetic relationship of *S. stercoralis* between dogs and humans remains unclear; haplotype A is considered zoonotic, whereas haplotype B has only been isolated from dogs (Ko *et al*., 2020). As *Strongyloides* DNA was detected in only 2 fecal samples here, it is likely that *Strongyloides* spp. may not be significant contributors to canine ill health in Grenada. Microscopic examination did not reveal larvae in any of the 232 samples, which is not surprising, given the limited fecundity of adult *Strongyloides* spp. and/or the tendency of larvae to hatch in the gut which are not readily detected using a conventional flotation-microscopy method. Each year, a number of human cases of hyperinfection syndrome of strongyloidiasis are reported by clinicians at the Grenada General Hospital. These cases may occur in patients with HTLV-1, as Grenada has the second highest known prevalence of this infection with immunocompromising virus in the world (Willems *et al*., 2017). Therefore, future studies – using a larger sample size – will be required to determine the prevalence of *S. stercoralis* in dogs in Grenada and to assess whether the zoonotic haplotype is present in this country.

*Ancylostoma caninum* was the predominant hookworm species found in Grenada (Zendejas-Heredia *et al*., 2022). The absence of *A. braziliense,* which is the most common cause of long lasting, serpiginous CLM, was surprising and needs further investigation, as the latter species appears to be rarer than the other hookworm species. This finding is consistent with the observation by local physicians that CLM is not commonly seen, despite a high infection rate of hookworm in dogs that roam frequently visited areas, such as the main tourist beach in Grenada (Schwartz *et al*., 2022; Neill, personal communication, Dec. 2009). The new molecular evidence suggests that *A. braziliense* may not be as widespread as previously thought, although additional studies of cats should be conducted, as felines can also serve as hosts (Bowman *et al*., 2010).

Of the *Toxocara* species, *T. canis* was confirmed as present in this population using PCR techniques and sequencing. While *T. canis* antibodies have been recently serologically detected in people in Grenada (Ziegler, 2022, unpublished results), no official reports of clinically significant infection have been documented (Schwartz *et al*., 2022). Implementation of prevention programmes for zoonotic STHs including *Toxocara* spp. in the canine population would likely help keep human clinical infection rates low.

Here, 14% of dogs positive for at least 1 STH showed polyparasitism. Roaming behaviour and BCS were determined to be significant predictors of test-positivity for STHs in dogs. Roaming dogs likely encounter numerous pathogens as they frequent many different properties and interact with numerous environments, other animals and people. Body condition is a parameter regularly used to assess health status in dogs by estimating fat deposits around specific parts of the body and is often recorded on the lower or skinnier end in dogs with parasitic infections (Fung *et al*., 2014). In this study, dogs with a low BCS were more likely to be infected with at least 1 of the gastrointestinal parasites investigated than dogs that were of optimal weight. A higher BCS was associated with *Toxocara* spp. infection, which may be attributable to the subjective physical assessment of pot-bellied puppies and pregnant dogs found to be infected.

Appropriate timing of anthelmintic treatment, especially targeting the prevention of transplacental/transmammary transmission for *Toxocara* and hookworm, would be an effective means of reducing parasitic transmission but requires funds to support veterinary care which is often limited in low- and middle-income countries. Such a strategy would reduce the imperative for owners to have their puppies treated within the first 2 weeks postpartum to prevent egg shedding in this highly infected population (Gates and Nolan, 2009). Emphasizing the importance of public education to reduce roaming behaviour in dogs, remove and dispose of canine feces and wash hands regularly are also important means of controlling transmission (Macpherson *et al*., 2022; Massetti *et al*., 2022; Walker *et al*., 2023). The presence of *A. ceylanicum* requires that further behavioural changes be adopted as well, such as wearing shoes outdoors and proper disposal of human and animal feces (Baker *et al*., 2018; Nery *et al*., 2019; Colella *et al*., 2021; Walker *et al*., 2023). Further studies of *Strongyloides* spp. in children and immunocompromised individuals are warranted, as well as studies of *A. ceylanicum* in people >12 years of age, as this demographic comprises >80% of hookworm-infected individuals in the tropics (Nutman, 2017; Colella *et al*., 2021).

In conclusion, the livelihoods of dogs in Grenada provide ideal conditions for parasite transmission and may possess the potential for zoonotic spread. This study stimulates future, larger-scale investigations of zoonotic parasitic helminths in animals and humans in Grenada and other parts of the West Indies using molecular diagnostic tools in order to elucidate their public health impact.

## Data Availability

Sequence data are available from GenBank, under accession numbers KU724129, MN076383, MK4686, AB923886, and LM525870.
